# An exploratory evaluation of a shared learning community of practice approach to improving gender equity in sports and physical activity programming

**DOI:** 10.3389/fspor.2026.1895135

**Published:** 2026-09-15

**Authors:** Julie McCleery, Ellyse Diamond, Briana Schulte, Lindsey Whitford

**Affiliations:** 1 Center for Leadership in Athletics, University of Washington, Seattle, WA, United States; 2 University of Vermont, Burlington, VT, United States; 3 Lakeside School, Seattle, WA, United States; 4 Soccer Without Borders, Seattle, WA, United States

**Keywords:** community of practice, gender equity, intervention, physical activity, shared learning, sports

## Abstract

**Background:**

This study explores the efficacy of a nine-month intervention that engaged twelve sport and physical activity organizations in a single U.S. county in shared learning, reflection, and problem-solving around gender equity. The Gender Equity Shared Learning Action Team (SLAT) was designed to address barriers to girls’ participation in structured sport and physical activity programs through a community of practice (CoP) approach. Participating organizations completed a Gender Equity Assessment, created Gender Action Plans (GAPs), and participated in shared learning and group discussion over the course of nine months.

**Methods:**

An evaluatory mixed-methods framework and collaborative grounded theory approach was used to evaluate this intervention, combining quantitative data from pre- and post-intervention assessments with qualitative data from semi-structured interviews.

**Results:**

Eight out of twelve organizations successfully completed the intervention. Descriptive pre- and post-assessment results showed observable increases in cohort mean scores for most gender equity practices, with the biggest increases coming in *training personnel* and *access to resources*. However, challenges such as staff turnover and lack of organizational capacity hindered full implementation of the GAPs. Interviews highlighted the value of the CoP approach, especially in building community and sharing knowledge but noted difficulties in organizational changes due to resource and staffing limitations.

**Conclusion:**

The SLAT demonstrated that a community of practice model can effectively support gender equity in youth sports and physical programming, though there are significant barriers in under-resourced organizations. Future interventions should focus on long-term investments in capacity-building to ensure sustainable organizational change related to gender equity.

## Introduction

The World Health Organization reports that 85% of female adolescents across the globe are not meeting recommended physical activity levels (1). Within the United States, compared to male youth, female youth tend to be less physically active with 23.1% of males and 10.2% of females meeting the most recent youth activity guidelines (2). In King County, Washington, only 24% of 8th girls compared to 36% of boys meet the CDC's recommendation of 60 min of physical activity daily (3). This trend continues as girls age, accelerating markedly in the teenage years when more girls, including girls who had been regularly involved in sport and exercise, disengage from sport and exercise due to a variety of complex, gender-specific barriers (4, 5). The disengagement of girls from sport and exercise, particularly in the teenage years, creates a significant missed opportunity for this part of the youth population, with impacts on their physical and psychological well-being. The positive physical impacts of exercise, including cardiovascular health (5, 6), bone strength (7), and the prevention of other noncommunicable diseases (8) are well known. Similarly, there is a demonstrated positive relationship between physical activity (through exercise and sport) and mental well being (5) and positive self-esteem among adolescent girls (5). Research shows that girls and boys who participate in at least one sport had 1.5–2.5 times lower rates of mental health disorders compared to those who do not participate in sport. When looking specifically at clinical depression rates in girls, those who participated in sport had an approximately 2.6 times lower rate than those who did not participate (9).

### Barriers to physical activity

Along with gender, there are a range of individual or family-level determinants related to physical activity and sport participation including socio economic status, language spoken at home, and race/ethnicity (10, 11). Youth from low affluence families must confront a number of environmental and structural barriers to physical activity participation including lack of space, unsuitable infrastructure, lack of equipment, program cost, and lack of transportation (11, 12). In a 2019 study in King County, WA, barriers faced include cost (13% of youth never participated in sport or organized activities due to cost; 11% dropped out due to cost), concerns about personal safety outside, inequity in access to sports programming and facilities, and transportation (13).

Additional sex and gender-specific barriers that impact girls’ participation in sport and exercise, and may contribute to their disengagement from sport and exercise, include menstruation (4), a lack of confidence and perceived competence (5, 14), competition (5), boys inhibiting girls’ active participation and play (5), decreased motivation (14), increased school work, not feeling safe exercising outside (4), as well as gender stereotypes (14), media representations (14), lack of role models (14), and a dislike for structured sports (10).

### Intervention domains

Due to these barriers that cross ecological domains, interventions targeting more than just the individual girl and/or her family are essential to increasing sport and physical activity access and engagement (15, 16). The socioecological model has been used to better understand and target the multilevel factors influencing girls’ PA (5, 16). This model takes interpersonal, organizational, social, and environmental variables into account for addressing PA gaps (16). This is especially useful in sport due to its gendered history and the way sociocultural gender norms o have shaped the youth sports and physical activity ecosystem as well as sports program leadership (17). The Sports & Fitness Industry Association found that 26% of youth sport head coaches were female in 2022, only up by 2% from 2019 (18). Further, women are under-represented in administrative, executive and board leadership positions in sports organizations (19). Gendered approaches are also often evident at the program level whereby organizations shape sports and physical activity approaches for boys without considering whether girls are also well served by these same practices. Working to make changes to programming therefore needs to be accompanied by addressing policies and practices related to hiring, budgeting, structures, and operations that have been shaped by a dominant masculine culture and ethos (19). Despite knowledge of the need for a change, and even increased investment (20), the pace of programmatic change for gender equity as been “glacial” (20).

### Communities of practice as intervention

One approach that has emerged to address gender equity across sports and physical activity at the programmatic level, while attending to the broader organizational and environmental framework, is the Communities of Practice (CoP) model. CoPs involve groups of people sharing knowledge and expertise about a concern or topic in order to deepen their understanding (19, 21). According to Culver et al., there are three dimensions of a CoP: mutual engagement, joint enterprise, and shared repertoire (21). The mutual engagement is characterized by social interactions that are informal, evolving, and ongoing (19, 21) and result in the development of shared knowledge and tools to face the challenges at hand (21).

CoPs are potentially efficacious for gender-based change in sport for a number of reasons. First, CoPs operate across levels and mediate across them. As Hanlon and Taylor note, a CoP “can purposefully support change and provide rich experiences to benefit participants and their sport organizations, individually and collectively” (19). Participants bring their individual knowledge and experiences into a shared social space, where collective meaning-making occurs, and then carry insights and tools back into their organizational and broader social contexts (19, 21). Second, as Culver et al. note, a CoP is defined not by formal organizational boundaries but by shared practice through which participants collectively examine, challenge, and reshape the everyday practices through which gender inequity is reproduced (21).

Recent examples, nationally and internationally, of gender equity, sports-focused CoPs range from those driven by researchers to National Governing Bodies to grassroots organizations (19, 22–24).

Culver et al. describe a case study of The Alberta Women in Sport Leadership Program, a multi-phased intervention that included a CoP for 12 sport leaders who also designed and implemented gender equity initiatives for their sport organizations, facilitated by six mentors. They reported mixed results due, in part, to capacity challenges of participants (22). Similarly, a recent project in Australia used a CoP approach to bring together eight CEOs of sports organizations to improve gender equity; the strengths of the approach included a supportive network, valuable conversations, and momentum building that led to an organizational push for change (19). A U.S. State Department-funded project in 2023 brought together 17 women educators in Pakistan for a professional learning community approach to support women's sport knowledge and engagement in sports leadership (24). Despite the increasing popularity of the CoP approach for gender equity in sport, no recent examples examine its use within the youth sports and physical activity realm (a seminal CoP in sport paper by Culver et al. examines CoP in youth baseball but not in the context of gender equity). This paper describes a mixed methods pilot evaluation of a grassroots CoP intervention that took place in one county in the United States. This CoP was called the Gender Equity Shared Learning Action Team (referred to as “Gender Equity SLAT” or “the cohort” throughout the remainder of the paper) and was characterized by a CoP of coaches and administrators from youth sport and physical activity programs focused on improving programmatic and organizational gender equity. This project provided an opportunity to conduct a pilot study of the impact of the Global Goal 5 (GG5) Accelerator model on a cohort of twelve youth sports organizations in King County. Named after the United Nations Sustainable Development Goal of Gender Equality, the GG5 Accelerator model takes an ecosystems approach to advancing gender equity through sport. In this model, direct service organizations join a regional cohort, and, alongside facilitators, work to understand the current state of gender equitable policies and practices in their organization; identify priority areas and commitments to focus on, and create gender action plans to accelerate progress toward their gender equity goals while supporting and holding each other accountable. GG5 was created through a collaboration between Soccer Without Borders (SWB), Women Win, and Common Goal—all organizations with decades of experience in the sport for development field internationally. Prior to its adoption for this pilot project in the United States, SWB had run the GG5 model with cohorts in five countries. This was the second cohort in the United States and the first to focus on all sports, in lieu of just soccer.

While the GG5 program had been running for a number of years, this project offered the opportunity to take a collaborative approach to understanding the intervention's strengths, challenges, and outcomes in this U.S.-based, youth-serving, multi-sport context. As such, the research team included a university-based researcher and research assistant, as well as an SWB employee with experience developing and implementing the program, and a community-based project manager who is also a coach. The research questions were 1) What are the strengths of the Gender Equity SLAT? 2) What challenges emerged during implementation? 3) What cohort-wide outcomes could be identified, and 4) What modifications should be made to the intervention to address challenges and enhance outcomes?

## Materials & methods

Since the goal of this pilot study was to explore the outcomes of an intervention designed to address, at the programmatic level, barriers to girls’ participation in structured youth sports and physical activity programs. An intervention mixed methods framework was used (25) along with a collaborative grounded theory approach (24). A grounded theory evaluatory approach allows researchers and stakeholders or participants to inductively develop insights from the data gathered during an evaluation to discover program outcomes or impact mechanisms as they emerge, rather than evaluating against predefined objectives (26).

The mixed methods evaluatory framework includes both quantitative data to understand the aggregate impact of the intervention and qualitative data collected to “understand contextual factors during the intervention that could affect the outcome and/or explain results” (25) and, in this case, help shape future interventions. Methods included pre-and post- intervention assessments and post- intervention semi-structured interviews with cohort members. The Institutional Review Board at the University of Washington considered the study exempt; nonetheless, the research team consented all interview participants.

### Participants

The participants in the Gender Equity SLAT cohort were recruited for the intervention from the King County Play Equity Coalition (KCPEC). The KCPEC is a collective action initiative, with over 130 organizational members dedicated to systems change, that increases access to sport, play, and outdoor recreation in the region. The opportunity to participate in the cohort was offered to any of the coalition's member organizations providing direct service to young people. The 12 organizations that participated represented a broad range of sports and activities, organization size, and population served (see Table 1 for detail). Some organizations were large, long-standing community organizations, like YMCA of Greater Seattle, that offer multiple sports and serve a broad swath of the population while others were relatively new organizations, like Washington Student Cycling League and First Five Years & Beyond, that offer one sport or activity or serve a specific population of youth.

**Table 1:** Participating organizations & associated activities.

Organization	Org Type	Primary population served	Major Gender Action Plan project
George Pocock Rowing Foundation	Rowing	High school students; boys/men & girls/women	Launch summer camp program for middle school girls of color
Parkour Visions	Parkour	All ages; all genders	Increase participation of girls across all programming; project not completed
Seattle United	Soccer	Ages 5–18; boys/men & girls/women	Improve entry level programming for girls in competitive program
Congolese Integration Network	Social Services	All ages; African Diaspora immigrants/refugees	Launch new soccer program for girls
Baseball Beyond Borders	Baseball/Softball	Ages 10–18; majority boys/men of color	Launch new softball program
First Five Years & Beyond	Early Learning	Families with children under 5	Launch physical activity program based on girls’ interests
YMCA of Greater Seattle	Multiple sports	All ages; all genders	Improve recruitment of girls for sports leagues
Washington Student Cycling League	Mountain Biking	High school students; primarily boys/men	Launch a girls’ only course preview ride prior to weekend races
DiscNW	Ultimate Frisbee	All ages; all genders	None completed
Shoreline Sports Foundation	Basketball	High school students; boys/men & girls/women	None completed
UPower	General movement	High school students; primarily in juvenile detention; primarily boys/men	Launch basketball program for hijab-wearing girls
Washington Education & Squash Academy	Squash	Youth; boys and girls	None completed

Each organization participating in the cohort was asked to designate one program administrator and one coach to participate in the Gender Equity SLAT. Participating organizations received $2,250 to support their engagement in cohort activities and to allocate towards supporting their internal change effort.

### Intervention design

The Gender Equity SLAT intervention was a modified version of the GG5 created by SWB, Women Win and Common Goal. Prior to this Gender Equity SLAT in the US, this intervention approach had been utilized in East Africa, Europe, Latin America and once in the US. The intervention has four main components, all of which are centered around regular cohort CoP meetings, intended to share challenges and best practices through each step.

1) Completion of a Gender Equity Assessment (GEA).

2) Development of a Gender Action Plan (GAP) by each organization based on organizational goals and the results of the assessment.

3) Implementation of the GAP by each organization.

4) Collective support and accountability for implementation through monthly meetings and shared learning activities with the broader community of practice.

The “collective support” topics were chosen based on the needs of the cohort identified through the GEA. The cohort-wide training sessions included coach recruitment, best practices for girls’ inclusion in programming, and inclusive recruitment practices (all described in greater detail below). Two facilitators, one from SWB and the other from KCPEC led the Gender Equity SLAT intervention.

### Gender equity assessment

All 12 organizations were asked to complete an organizational self-assessment, the GEA. The GEA, like the overall intervention was developed through the collaboration between Women Win, Common Goal, and SWB. Women Win had originally developed the assessment as a gender inclusion self-reflection tool. When the three organizations partnered to run the first GG5 program in East Africa in 2020, they jointly evolved the language in the self-reflection tool to focus on soccer and added specific sports content to bring the gender inclusion targets to life using their expertise in running global gender-focused sport for good programs. The tool was tested and iterated over two years based on field testing and feedback from participants and intervention facilitators; the adjustments focused on both clarifying content and enhancing accessibility for participants.

The current instrument consists of questions specific to an organization's approach to gender inclusion and gender equity across three organizational levels: *On the Field*; *On the Sidelines*, and *In the Organization.* Each broad category is made up of between 5 and 8 sub-categories, which are each made up of 1–5 items (see Table 2). *On the Field* items generally measure the degree to which individual girl participants are being supported to join, participate, and are having their needs met through the program. *On the Sidelines* items generally measure the degree to which coaches, specifically women coaches and those that coach girls, are supported to enhance the experience of girl participants and are, in turn, supported and retained by the organization. *In the Organization* items generally measure the degree to which policies, procedures, and budgets are oriented towards supporting girls programming and women's leadership (see Table 3 for sample questions). The aims of the GEA were 1) to evaluate the organization's approach to gender inclusion and gender equity prior to the intervention; 2) to provide each organization with information about current practices that would inform their choices for creating a Gender Action Plan (GAP), and 3) to evaluate the organization's approach to gender inclusion post intervention.

**Table 2:** Levels, categories, and subcategories of the GEA instrument.

On the field	
Subcategories	Items
Recruitment	Recruitment plan
	Actie recruitment
	Multiple entry points
Barriers to regular participation	Economic and time restraints
	Adequate equipment
	Appropriate apparel
	Menstruation management
	Transportation/travel
Sport quality program	Coaching
	Competitions
	Skills development
	Team size
	Program accommodations
Safe spaces	Quality playing space
	SAFE activity spaces
	Mixed gender activities
	Toilets/changing rooms
	Safeguarding policies
Girls leadership	Leadership roles
	Role models
	Decision making
	Cultivating leaders
	Girl-led
	Promotion
Community of support	Attitudes of boys & men
	Embeddedness
	Advocates
Continuous learning	Access to data
	Participant information
	Understanding exits
	Youth survey & feedback

On the sidelines	
Subcategories	Items
Female coaches	Coach recruitment
	Female presence
	Equal coaching opportunities
Access to resources	Programming resources
	Equal renumeration
	Coaching apparel
Coach leadership	Leadership skills
	Role models
	Coach development
	Visibility
Coach training	Technical training
	Gender sensitive coaching
	Support & feedback
Coach monitoring	Tracking coaches

In the organization	
Subcategories	Items
Gender strategy	Gender inclusion
	Gender equity
	Visibility of Female Leaders
Gender policies	Gender Inclusion Policy
	Equal Opportunity Employment
Training of personnel	Gender Training
	Advancement Opportunities
	Advice
Financial resources	Balance of Financial Resources
Communication & advocacy	Gender Equity in Media
	Communications Training
	Gender Sport Advocacy
Culture & mindset	Gender Equity Support
	Positive Discrimination
	Grievance
	Willingness to Change

**Table 3:** Examples of questions from gender equity organizational assessment.

On the Field—Program Design and Implementation		
Subcategories	Items	Sample Scoring Guidance (Scoring 1–5)
Recruitment	Recruitment Plan: Our program has an effective, ongoing strategy in place that specifically targets girls for recruitment into the program, so it serves girls at full capacity.	Plan sets a clear goal for who and/or how many girls the plan aims to recruit Plan details how to reach marginalized or hard to reach groups such as out-of-school girls, girls with disabilities and girls from ethnic minorities Plan is informed by girls from the group with an active role defined for girls to lead In a strong program, girls are excited to invite others to the team and become a part of the recruitment plan
	Active Recruitment: Our program outreaches to potential new girl participants through experiential actions and activities, inviting the potential participants to experience some of the culture of the program or gain skills to support their entry prior to formally joining.	Holding a girls’ community clinic to share a basic skills of the sport and invite girls to formally join the program Adding an element of your usual practice session (opening, team-builder, game) to outreach presentations at schools or community fairs Following up with referrals to community members by speaking with parents/caregivers about the benefits of and concerns about sport participation and ensure the safety of girls
	Multiple Entry Points: Our organization offers multiple and diverse avenues through which girls can find out about the program and sign up.	Holding a special event for younger siblings of current participants, so they can look forward to aging into the program Having a shorter season for young girl participants, so they can develop a connection to sport before full participation Sharing program information with community resources and locations frequented by girls (schools, churches, health centers) Seeking referrals from agencies that work closely with girls or their parents/caregivers (refugee resettlement agencies, school counselors, community leaders) Hosting events that are open to the entire community or specifically engage a portion of the community not currently represented in the program Current participants and families are encouraged to invite their friends to join

To complete the assessment, participants rated themselves on a scale of 1–5 on each of the 64 items. A one was defined as Very Weak/Rarely/Foundation (an organization is at the beginning of the gender inclusion journey in this area and does not currently have anything in place), and a five was defined as Thriving/Always/Maintaining and Improving (an organization is using best practices on this target and has a constant eye towards quality improvement). Teams were given three weeks to complete the assessment and were asked to engage in conversation with other staff and board members in their organizations to come up with ratings. Data was self-reported through Google Sheets which aggregated the items into subcategory scores for each organization and aggregated subcategory scores across all organizations for a cohort-wide score on all items and subcategories. After completion of the intervention, organizations rated themselves on the assessment items a second time using the same methodology. A portion of the assessment is provided in Supplementary Material 1. The primary adaptation made to the tool by this research and intervention team was to adjust language to be inclusive of all sports, not just soccer.

### Intervention implementation

The full intervention was successfully implemented by eight of the initial 12 organizations. During the course of the nine months, one organization completely closed and three others experienced staff shortages or transitions that made participation challenging and resulted in incomplete adherence to the full intervention protocol. The eight organizations that completed the intervention prepared and implemented a Gender Action Plan (GAP) informed by the GEA and facilitated by the activities in the intervention and a workbook. Table 1 provides a brief overview of each organization's main GAP topic. An example of a full plan, which includes detailed descriptions of four priority areas, can be found in Supplementary Material 2. Achievement of these GAPs were mixed; at the final session, organizations reported some of the challenges that prevented them from achieving their goals. While many of these challenges are addressed in the interview results section below, one of the primary challenges mentioned across the organizations was lack of broader institutional buy-in to the GAP implementation.

### Community of practice & training schedule

To support building knowledge and assist with GAP implementation, the cohort met for shared learning sessions. Some of these sessions consisted of topical trainings determined by organizational needs and others were dedicated to CoP discussion about various aspects of the intervention. See Table 4 for a timeline of the sessions.

**Table 4:** Timeline of sessions.

Date	Activity
March 1, 2022	Gender Equity Assessment Introduction & Discussion
April 5, 2022	Gender Action Plan presentations
April 26, 2022	Gender Action Plan progress discussion
June 1, 2022	Topic: Barriers and facilitators to recruiting & maintaining women coaches
July 11, 2022	Topic: Inclusive Coaching Best Practices
September 12, 2022	Topic: Inclusive Recruiting Best Practices
October 24, 2022	Closing discussion on challenges, successes, and learnings
December 15, 2022	Presentation of learnings to full Coalition

### Learning session topics

#### Coach recruitment

This meeting consisted of a discussion focused on best practices for recruiting women coaches. Using local data, the cohort did a mapping exercise of barriers experienced by session participants when recruiting women coaches. The session also included a case study discussion about organizations building pipelines and training for women coaches. Finally, cohort members brainstormed ideas to build the community of women coaches across King County in order to reduce social isolation experienced by women coaches.

#### Best practices for girls’ inclusion training

This 4-h, on-field session trained coaches and organizational leaders on best practices for coaching to keep girls in sports programming. The primary best practice themes shared were contribution matters; confidence comes from competence; celebrate big and small wins, and share power. For each of these practices, coaches and organizational leaders participated in experiential learning via on-field games that demonstrated the best practice and created opportunities to debrief and brainstorm on how to bring these practices into their programming.

#### Girls’ recruitment

The final topical session focused on recruitment and outreach activities for girls. Participants walked through a series of questions designed to help coaches and administrators reflect on the ways their outreach and initial contact with girls could be improved to enhance inclusion and retention. The questions were adopted from a Breaking Barriers Academy online training module entitled Recruiting Inclusive Sports Talent That Stays (27), which focuses on ideas like passive vs. active recruitment, recruitment goal setting, and understanding your community to develop targeted messaging. Organizations shared their best practices and challenges with one another.

The remaining sessions allowed for orientation, group discussions, information sharing and conversation about the gender equity assessment, the gender action plans, and overall successes and challenges.

### Interviews

Interviews were conducted at the conclusion of the project with all organizational leaders and coaches who participated in the Gender Equity SLAT. The semi-structured interviews were conducted on Zoom, lasted approximately 1 h, and were recorded and transcribed. Questions, which were developed by the research team and informed by the research aims and questions from similar studies (21, 22) focused on 1) their role in designing and implementing the Gender Action Plans for their organization or target program; 2) their perceptions of challenges, barriers and facilitators to successful implementation; 3) their general thoughts about the SLAT process, and 4) their general thoughts on the GEA tool and process (see Supplementary Material 3 for the Interview Guide).

### Analysis

The data from the intervention, GEAs, and interviews were analyzed separately per concurrent data analysis procedures for a triangulation mixed method design (28). The pre intervention GEA data was entered by each organization, based on their self-assessment, into linked spreadsheets that automatically populated to a master file that showed aggregate scores for the cohort in each subcategory. The project manager supported organizations to ensure any missing data was addressed, and she did manual checks of the master spreadsheet to ensure data accuracy. This process was repeated for the post-GEA self-assessments.

Due to the limitations of the collected data, and the goals of the evaluation, descriptive analysis, as presented in Table 5, of the aggregate cohort subcategory means was deemed to be the most appropriate method to compare pre and post intervention outcomes. Given the exploratory and grounded nature of the evaluation, our aim was not to test a confirmatory hypothesis but to examine observable patterns that could inform future interventions and evaluatory processes. As such pre–post differences were examined descriptively using observed changes in means and magnitude of change (19, 24). No inferential statistical tests were conducted. Limitations of this analytic approach are further discussed in the limitations section.

The interviews were first inductively thematically coded through a series of stages by one member of the research team to make sense of participant experience (29). The stages included reading through each transcript for data familiarization, coding, and theme development based on the codes (24). The coding was supported by ongoing analytic memoing to track conceptual development throughout the coding process (29). The initial themes and memos were then shared with the full research team which included the SLAT facilitator and the project manager The team discussed the application of the emerging themes in relationship to the research questions and compared them with their notes and observations from the implementation and activities with which they were directly involved. This confirmatory process identified areas for deepening the emerging categories, which were then refined through a second round of coding (29). These themes were again discussed by the team to challenge assumptions and strengthen the confirmability of the final themes (24, 25). The quantitative and qualitative data are presented separately in the results section with the discussion section being the primary place of analytic convergence (23) and data integration (25).

**Table 5:** Gender equity assessment scores across categories and subcategories.

On the field			
Sub-category	Cohort pre-measure	Cohort post-measure	Change
Recruitment	2.37	2.71	+0.34
Barriers to participation	3.00	3.37	+0.37
Sport program quality	3.58	3.57	+0.01
Safe spaces	4.00	3.74	−0.26
Girls leadership	2.99	3.05	+0.06
Community of support	2.96	2.98	+0.02
Continuous learning	2.30	2.57	+0.27
In the organization			
Gender strategy	3.53	4.05	+0.52
Gender policies	2.75	3.36	+0.61
Training of personnel	2.50	3.40	+0.90
Financial resources	4.50	4.43	−0.07
Communication and advocacy	3.53	3.71	+0.18
Culture and mindset	4.40	4.50	+0.1
On the sidelines			
Female coaches	3.55	3.90	+0.35
Access to resources	3.43	4.21	+0.78
Coach leadership	3.30	3.78	+0.48
Coach training	3.22	3.88	+0.66
Coach monitoring	1.80	2.13	+0.33

## Results

### Result 1: gender equity pre & post assessment

The aggregate mean scores from the GEA pre-measure consist of data from 10 organizations while the post measures consist of scores from 7 (of the 8) organizations that completed the intervention. In the pre-intervention assessment, the lowest aggregate rating was given to the sub-category *Coach Monitoring*. The only item in this subcategory includes “tracking coaches” and asks the degree to which an organization collects and utilizes data and information about coaches, including data disaggregated coach by gender; data on why female and male coaches leave the program, and taking action in response to patterns that emerge from the data. The highest rating was given to the *Financial Resources* subcategory which has only one item: “balanced financial resources.” This includes balanced resources between boys’ programming and girls’ programming including coach salaries, equipment, and field time, etc.; with additional resources allocated as necessary to enable girls’ participation.

Across the 18 subcategories (which are built on 64 total items), in the three organizational levels, the observed change in the aggregate score was positive in 16 of them (see Table 5). The observed change was negative in two categories: *Financial Resources* (−.07) and *Safe Spaces* (−.26) (which is defined as quality playing space, safe activity spaces, mixed gender activities, toilets/changing rooms, and safeguarding policies). The greatest gains were in *Training of Personnel* (+.9) and *Access to Resources* (+.78).

### Result 2: interview findings

Interviews with nine organizations (a total of 14 people) identified four major themes. Two themes that can be considered strengths of the intervention—Organizational Knowledge and Intention and Increasing Collaboration. Within Organizational Knowledge and Intention, three sub-themes were identified: Recognizing Blind Spots, Becoming More Intentional, and Replication. Two other themes emerged that can be considered challenges: Staff Turnover and Lack of Organizational Capacity. Each theme is discussed in this section.

### Staff turnover

Regarding staffing challenges, a number of organizations, of varying sizes, had participating coaches leave the organization during the course of the intervention. Others had staff members who were central to implementation shift to other roles. One program leader said, “one of the challenges was…retaining the number of people that started off in the project in terms of staff on my end.” Another program leader noted the coach she was working with left her role during the project which meant she had to work on the project alone and “that's really where the drop off was.” One program had enlisted a board member to attend meetings to help with any necessary policy-level learning or implementation and after two meetings he stopped attending and said he got too busy at work to continue supporting the project. This turnover made it difficult for some programs to sustain their momentum within the cohort and limited organizational knowledge-building and policy change due to lack of sustained engagement with the cohort activities and execution of the GAPs.

#### Lack of organizational capacity

Similarly, several organizations referenced a lack of capacity to implement the desired changes that went beyond staff turnover. One program leader said their organization was still bouncing back from the challenges of Covid and noted their main limitations were “capacity, capacity and time, time, time.” Two other organizations, both relatively new, said that implementing the gender equity changes while also running the organization proved challenging. One organization was trying to make changes while “in the heart of racing season,” with just two staff members, which turned out to be too much to do at once. The other organization said that the timing of the CoP was such that they were building their program alongside this work, and that was logistically difficult. This was a challenge expressed primarily by smaller organizations.

Even if programs experienced challenges and did not achieve all of their goals, many of them had positive experiences specifically with the community-building aspect of the project as well as the knowledge they gained from the GEA Tool.

#### Strengthening organizational knowledge & intention

Regarding the GEA tool, participants noted its value even as they described the process of completing it as “tedious” and “overwhelming.” A number of participants described questions as hard to answer because either they didn’t have a holistic enough view of the organization and organizational policies, or they didn’t have the positional authority or capacity to engage executive-level staff or the board. One participant in a larger organization said, for example, that her answers were skewed “knowing that we were doing a girls specific program…so, of course it's girls oriented, but other parts of the organization…I don’t know as much.”

Despite these challenges, participants found the GEA extremely helpful. One program leader said, “The first assessment especially it was, like, a little eye opening… I think it was really helpful, kind of pushed us in the right direction to be able to decide what we really wanted to focus on.” Another participant commented that it allowed her to think about the nature of their programming and “are we providing programming that is appropriate for them and fair to them and representative of their identities as they’re coming into this space.” Another noted that it “really challenged us to think about our program critically.” Even though many organizations came in with an orientation towards and an interest in improving gender equity within their organizations, they were surprised by their own blind spots and with the degree of knowledge-building and learning they still had to do.

Many participants noted that the knowledge they gained through the assessment helped them approach their girls’ programming with more intentionality. One program leader said, the assessment “created so much opportunity for us to think about how we were setting up the foundation of the program.” Another said it forced them to think about whether the program elements were just “part of the program” or if they were intentionally put there to enhance girls’ experiences. A third said, similarly, that while they expected to be “fives across the board,” they found that “even within a girl's focused, girls only program,” they still had work to do. The tool—and the intervention—gave organizations an opportunity to question structures and practices that they previously had taken for granted as “parts of the program” without consider their intention or value for the girls they were serving.

Lastly, several organizations talked about taking some of the knowledge gained and using it to replicate specific activities offered in the shared learning CoP monthly meetings. For example, one large organization replicated the recruitment and retention activity with staff at all of their branches “so they can start thinking about what can they do specifically to try to be more welcoming for females.” This ripple effect of knowledge sharing also occurred between peers and colleagues with participants commenting about the number of conversations they had within their organization, even with those not directly involved in the project. These replications and conversations suggested a measure of knowledge sustainability.

#### Increasing collaboration

Participants also identified the collaborative nature of the CoP as a strength. They appreciated both the community-building and networking aspects of the project as well as the idea generation and knowledge-sharing made possible by the community of practice. Participants expressed feeling both inspired and supported talking about how they all were “running up against the same types of issues and struggles.” One participant said,

“I feel like just being in conversation with other organizations on this project…is so helpful. I definitely love feeling very involved in things and I loved feeling involved in the project and like getting to know these other coaches and program administrators from various different (programs).”

A number of participants noted that the depth of the work solidified a type of relationship that went beyond meeting someone who is doing the same work to “creating actual relationships” that would extend beyond the life of the project, again pointing to a measure of sustainability in both the gender equity efforts and the networks built in service of those efforts

## Discussion

This study explores the efficacy of an intervention aimed at increasing girls’ participation and gender equity practices, more broadly, in structured youth sports and physical activity through a community of practice, program-level intervention. The assessment suggests some merits of collaborative approaches to addressing gender equity in youth sports programming and also reveals significant challenges with this CoP approach. Taken together, the qualitative and quantitative results, along with the completion rates from the intervention, suggest an uneven-ness in the efficacy of the intervention. This mixed picture is consistent with research about gender equity programmatic interventions in general (20) and CoPs specifically (19, 21, 24, 29).

The observed positive trends in the pre-post GEA results are offset by the fact that only 10 of 12 organizations completed the pre GEA and only 7 of those organizations completed the post GEA. Those score increases, therefore, represent only those organizations who had the capacity to complete the intervention and the GEA. The interviews confirm the challenges with completing the GEA, suggesting it was time consuming and confusing, and that program participants weren’t always in a position to answer questions that were not at their “level.”

Future iterations of the GEA should reduce both participant burden and the number of assessment categories to work towards developing a tool that delivers similar insights with increased reliability and functionality for measuring organizational change. Further, the instructions related to GEA data collection might specify the stakeholders necessary for reliable completion and provide a suggested structure through which to engage those stakeholders.

Regarding the intervention implementation, only 8 of the 12 organizations in the Gender Equity SLAT cohort were able to fully complete the intervention, and the CoP meetings were not always consistently attended by the same coaches or organizational leaders. One organization closing during the intervention and some smaller organziations struggling to run programming and commit to the intervention may point to a challenge specific to grassroots, youth-serving organizations in the sport and physical activity space. Even for those organizations that completed the intervention, interviews explicated how challenges with staff turnover and organizational capacity contributed to inconsistency amongst the cohort with attendance and GAP completion.

This challenge is not unique to the Gender Equity SLAT cohort. High rates of coach turnover are the norm in youth sports, creating a transient and dynamic sector (30). Further, the inherent instability of coach and non-profit staff salaries means that retaining people long enough to make deep organizational change is difficult. Similarly, one of the main challenges of the Alberta Project was participation and retention which created a lack of consistency with the CoP, and which challenged the “development of connection within the community” (21).

Participants also reported mixed success in the implementation of their Gender Action Plans, the specific programmatic changes they had chosen for their organizations. Interview findings suggested that implementing a structural change or even a programmatic change while directly serving youth was a challenge for all participants. Previous research into CoPs in sport has found similar challenges related to organizational capacity for change in the perennially under-resourced non-profit sector. For example, a 2018 study exploring the organizational capacity of three National Sport Organizations in Canada to implement gender equity initiatives aimed at increasing the participation of women in sports found there to be human resource and financial challenges very similar to those found in this project including lack of staff time, lack of resources to tackle long term planning, and a need to focus on day to day work in lieu of programmatic and organizational change (31). This challenge is particularly problematic for organizations that have been set up to serve both boys and girls but are utilizing structures, policies, and practices that do not necessarily take the needs of girls and women into account in the formative stages of the organization. Without being able to find the time and resources to make a sustained commitment to deep organizational change, systemic gender inequities will be perpetuated (19).

Interview findings suggest the major benefits of the CoP approach for the Gender Equity SLAT included community building, shared problem solving and knowledge building. The knowledge building and shared problem solving likely contributed to the positive trends in post-GEA assessments for those who were able to complete the project. Participants described the intentionality they were able to bring to their programming based on new insights from the GEA and shared learning. This intentionality likely resulted in changes and adjustments to policies and practices. These benefits are consistent with the literature about CoPs in sport which describe building knowledge (19), shared tools, routines, and ways of doing things (21) as an outcome. In the CoP of Australian sport CEO's, participants spoke about the ways increased knowledge created a sense of confidence that helped with other decisions related to organizational change for gender equity (19).

Based on the CoP model, what makes this new knowledge and intentionality become sustained organizational change, however, is the fact that it was built through community and is a reflection of the community's “mutual engagement” in the creation of new meaning (21). Willem et al, for example, note that shared learning in the sports-based youth development sector may be uniquely successful due to participants’ mutual engagement in a community dedicated to making sport as accessible as possible and a shared understanding of the associated challenges (23). SLAT participants similarly shared that community building around common “issues and struggles” as well as a common commitment to gender equity was a strength of the intervention. The relationships, and making of shared meaning with others who were similarly situated, were central to sustained participant engagement and learning. To the extent that the positive pre-post trends represented organizational engagement and change for the 7 organizations, those may have been mediated by the individual participants staying in relationship with others through the course of the intervention. This reflects the ways in which the CoP is intended to operate simultaneously at the individual, group, organizational, and cultural levels in pursuit of change (21).

Based on the findings from this exploratory evaluation and the existing literature, several recommendations can be made for future CoPs and similar interventions. First, any organizational-level intervention designed to attract more girls into a physical activity and sports program needs to account for the work of retrofitting or redesigning programs that weren’t designed with the needs of girls in mind. This deep work, whether supported by a CoP or other approach, is challenging given capacity limitations of non-profit organizations and transience of staff in the sport and non-profit sectors, espcially within youth-serving organizations. Funders and policy makers (sport NGBs) interested in shared learning and CoPs should prioritize long term, substantial funding and resources that allow organizations to deeply address the unique barriers faced by girls while simultaneously running programming. As much as a CoP approach builds community and produces shared knowledge, structural change, ultimately, is not work that can be done by only a few within an organization in a year's time. Finally, there is a need for more studies that investigate other types of program-level interventions aimed at increasing gender equity within sports and physical activity-based organizations since the gap between boys’ and girls’ sports and physical activity engagement is tenacious (32).

### Limitations

This was a pilot evaluation of a grassroots intervention conducted in one region of the US with a small sample size. Results are not generalizable to other regions or to other types of gender-equity focused interventions. The aggregate descriptive data represents only ten of 12 organizations for the pre-GEA and only seven organizations for the post-GEA; therefore, the score changes are not reflective of a matched pre and post set of organizations. Paired *t*-tests of pre and post subcategory scores of the seven organizations that completed the GEAs would provide more targeted information about those organizations’ changes. The descriptive approach taken in this paper comparing the observed pre and post cohort aggregate means allows for a broad look at cohort patterns consistent with the exploratory approach of this evaluation. Future evaluatory approaches should consider inferential analysis of each organization's pre and post GEA scores.

## Conclusions

Researchers utilized an evaluatory mixed-methods framework to explore the effectiveness of a pilot community of practice program-level intervention to improve gender equity in sports and physical activity programs using a grounded collaborative approach. While the pre and post assessments show that organizations who completed the intervention generally made progress, becoming more attuned to the need for gender-equitable policies, processes and programs, the implementation of the GAPs were inconsistent. The interviews also pointed to the strengths and challenges inherent in a CoP, program-level approach. The primary take-away is that inherent instability of youth-serving non-profit and sports-based organizations creates challenges for the type of organization-wide investment necessary for broad scale organizational change or adoption of new initiatives.

## Data Availability

The datasets presented in this article are not readily available because all data is identifiable and therfore not available to the public. Requests to access the datasets should be directed to Julie McCleery, juliem4@uw.edu.
