# Supplementary material for: An exploratory evaluation of a shared learning community of practice approach to improving gender equity in sports and physical activity programming
Source: Front Sports Act Living. 2026 Sep 15;8:1895135. doi: 10.3389/fspor.2026.1895135 (PMC13619867; doi:10.3389/fspor.2026.1895135)
Supplement: Supplementary file 1 [file Datasheet1.pdf]

Global Goal 5 Accelerator Gender Inclusion Self-Assessment

To learn more about this tool or for support with implementation, contact Soccer Without Borders at info@soccerwithoutborders.org

Introduction to the Global Goal 5 Accelerator:

According to the United Nations, “insufficient progress on structural issues at the root of gender inequality, such as legal discrimination, unfair social norms and attitudes, decision-making on sexual and reproductive issues and low levels of political participation” has impeded progress toward Global Goal 5.

Sports has a powerful influence on norms and attitudes at an individual, community, and global level. Participation in sport gives girls an opportunity to understand their bodies as athletes, build their confidence and voice as leaders, and access a community of supportive peers and mentors. The combination of these individual and collective impacts makes sport for good programs and organizations uniquely positioned to contribute to Global Goal 5.

The Global Goal 5 Accelerator aims to create concrete strategies, resources, and roadmaps in every region, providing tools, financial support, and accountability to elevate our collective efforts. Together we can:

1. On the pitch: Increase girls’ participation in participating in sport for good programs
2. On the sidelines: Ensure that every girl participant has a female coach role model in their community
3. In organizations: Ensure more gender-equitable organizations, including representation in leadership
4. In the Media: Raise awareness and understanding of football-related Global Goal 5 stories that aim to change perceptions locally and globally by spotlighting stories from the accelerator and its collective impact.

Gender Inclusion Self-Assessment & Gender Action Plan

This self-assessment is designed to help you think about various aspects of gender inclusion and how they relate to your organization and your sport programs. You can use it as a tool to promote dialogue about gender inclusion with your team and participants in your programs. The self-assessment focuses on three levels – in organizations, on the sidelines and on the pitch – and aims to help an organization to:

1. Assess where it is currently, providing a baseline to work from
2. Identify strengths to build on and share with other cohort members
3. Identify focus areas that need improvement

	4. Plan to take action on these focus areas						
	5. Set goals and targets to measure progress.						
This document becomes your organization’s Gender Action Plan. Your Gender Action Plan is a document that is a tool for your organization. It is not intended to be a comparison with others, nor a listing of required standards. It is most beneficial when the assessment is honest and thorough, and the goals are “SMART” (specific, measurable, achievable, realistic and time bound). You plan is both a snapshot of a moment in time and a living document. It captures quantitative data as well as future plans, which can be constantly evolving over time.<br><br>All organizations involved in the Accelerator will develop a Gender Action Plan with explicit commitments for each of the three levels to work towards in the spring, summer and fall of 2022.							

Self-Assessment Scoring & Scale

Three sections make up the self-assessment. Each section contains a number of focus areas that broadly describe issues related to gender inclusion. Each of the indicators comprises a variety of criteria that you score/rate your organization's performance on. Finally, there is space for a description, where you can include some brief points about what your organization has done in each focus area in order to justify the scores you have given.

Below is the scale you can use to indicate where along the gender inclusion journey your organization is. Please read each gender inclusion criteria carefully and then rate that criteria based on the scale below. Each criteria includes a short description and some also have examples to help you score/rate how your organization is performing on those criteria.

1: Very Weak/Rarely/Foundation level	
	Quality is very low and needs significant improvement
	Target is rarely or never met
	Your site is at the beginning of the gender inclusion journey in this area and does not currently have anything in place
2: Weak/A little bit/Early stage–	
	Quality is sufficient and meets the target in a basic way
	Implementation is somewhat consistent; more often than not
	Your site has started to implement with basic tools and resources
3: Average/Somewhat/Basic Implementation	
	Quality is sufficient and meets the target in a basic way
	Implementation is somewhat consistent; more often than not
	Your site has started to implement with basic tools and resources
4: Strong/Mostly/Strong Implementation	
	Quality is high, meeting the letter and spirit of the target and you have a way of measuring effectiveness
	Implementation is mostly consistent day to day/week to week; you can count on this happening
	Your site is actively implementing and significant thought and resources having been dedicated to this area
**5: Thriving/Always/Maintaining and Improving**	
	Quality is extremely high, going above and beyond the letter and spirit of the target, the program measure effectiveness
	Implementation is consistent day to day, week to week and from person to person, remains steady across transitions (seasons, people, etc)
	Your site has formed best practices on this target that can be recycled from year-to-year and there is a constant eye towards quality improvement

**Analysis**			
After self assessments are completed, the Shared Learning Team will share back tools and resources to analyze results.			

Coaches must be included in the completion of this portion

On the Pitch – Program Design and Implementation

What – Getting girls in the game means directly recruiting and retaining them in our programs. This section looks at recruitment, barriers, program design, safety, and infrastructure that lead to better access, engagement, and inclusion.

Why – It is important that gender inclusion is not only considered an administrative responsibility related to policies and procedures. Gender inclusion must be present in our actions on the pitch and behind the scenes.

Score each target thinking specifically about the program or site that your organization is focusing on for this progect, marking the target with a 1-5.

**1.1 Recruitment of Girls**		
**(a) Recruitment Plan & Goal**	**Score (1-5)**	**Notes and Comments**
Our program has an effective, ongoing strategy in place that specifically targets girls for recruitment into the program so it serves girls at full capacity.<br>**Examples**<br>- Plan sets a clear goal for who and/or how many girls the plan aims to recruit<br>- Plan details how to reach marginalized or hard to reach groups such as out-of-school girls, girls with disabilities and girls from ethnic minorities<br>- Plan is informed by girls from the group with an active role defined for girls to lead<br>- In a strong program, girls are excited to invite others to the team and become a part of the recruitment plan		
**(b) Active Recruitment**	**Score (1-5)**	**Notes and Comments**

Our program outreaches to potential new girl participants through experiential actions and activities, inviting the potential participants to experience some of the culture of the program or gain skills to support their entry prior to formally joining.<br>**Examples**<br>- Holding a girls' community clinic to share a basic skills of the sport and invite girls to formally join the program<br>- Adding an element of your usual practice session (opening, team-builder, game) to outreach presentations at schools or community fairs<br>- Following up with referrals to community members by speaking with parents/caregivers about the benefits of and concerns about sport participation and ensure the safety of girls		
**(c) Multiple Entry Points**	**Score (1-5)**	**Notes and Comments**
Our organization offers multiple and diverse avenues through which girls can find out about the program and sign up.<br>**Examples**<br>- Holding a special event for younger siblings of current participants, so they can look forward to aging into the program<br>- Having a shorter season for young girl participants, so they can develop a connection to sport before full participation<br>- Sharing program information with community resources and locations frequented by girls (schools, churches, health centers)<br>- Seeking referrals from agencies that work closely with girls or their parents/caregivers (refugee resettlement agencies, school counselors, community leaders)<br>- Hosting events that are open to the entire community or specifically engage a portion of the community not currently represented in the program<br>- Current participants and families are encouraged to invite their friends to join		
**1.2 Barriers to Regular Participation**		
**(a) Economic and Time Constraints**	**Score (1-5)**	**Notes and Comments**
Our organization's activities take into account girls' unique economic and time constraints within the family and community.<br>**Examples**<br>- Home commitments such as household chores and family responsibilities<br>- School commitments including attending regular classes, completing homework and studying for exams<br>- Lack of financial resources to participate<br>- On mixed gender teams, girls are not unfairly penalized for missing trainings or programming due to unique constraints		

(b) Adequate Equipment	Score (1-5)	Notes and Comments
Our organization provides girls with adequate equipment corresponding with their age, level of ability, and competition needs.<br>**Examples**<br>- Equipment for girls is comparable to what boys of a similar ability and age have<br>- Girls have female specific equipment, if applicable<br>- Adapted equipment is available for girls with disabilities if required		
**(c) Appropriate Apparel**	**Score (1-5)**	**Notes and Comments**
Our organization ensures girls have access to appropriate apparel for the cultural context as well as the level of competition they are engaged in.<br>**Examples**<br>- Apparel for girls is comparable to what boys of a similar ability and age have<br>- If required, uniforms or apparel are modified based on cultural and religious considerations specific to girls<br>- Girls have access to appropriate underwear including sports bras that fit<br>- Girls have access to uniforms that comfortably fit their body shape and size		
**(d) Menstruation Management**	**Score (1-5)**	**Notes and Comments**
Our organization acknowledges that girls' menstruation impacts their comfort and safety while participating in sport and ensures that girls are both comfortable and safe while menstruating during the sport program.<br>**Examples**<br>- Girls have access to sanitary napkins/tampons<br>- Girls and female coaches have a clean and private place to change or dispose of their sanitary napkins/tampons<br>- Girls have at least one female coach to comfortably talk with for suppor		
**(e) Transportation/Travel**	**Score (1-5)**	**Notes and Comments**

Our organization provides or accounts for safe transport options for the girls to get to and from activities<br>***Examples***<br>- Access and safe travel options are considered when selecting activity spaces (practice sessions, games, life skills, special events)<br>- Transportation plans are discussed with girls and their families as soon as a girl signs up for the program<br>- If/when necessary, girls are provided with safe transport to/from the field including but not limited to walking groups with other girls and vehicle from our organization<br>- If transport becomes unsafe due to weather, political activity, etc., activities are paused until new plans can be made or transport becomes safe again		
**1.3 Sport Program Quality**		
**(a) Coaching**	**Score (1-5)**	**Notes and Comments**
The head coach is a dynamic female role model that the girls trust, respect, and respond to.<br>**Examples**<br>- The coach has basic competency in the sport<br>- The coach has the necessary technical coaching ability to teach the girls in the group<br>- The coach has facilitation skills that create an inclusive environment that accommodates individual differences and enables all group members to experience success<br>- The coach shares power with the girls, inviting their feedback and voice<br>- The coach is a magnet; the girls are drawn to activities because they want to learn from her		
**(b) Competitions**	**Score (1-5)**	**Notes and Comments**
Our organization prioritizes providing girls with the opportunity to take part in organized matches and tournaments at various levels (intra-organizational, local, regional, national and/or international) as much as possible.<br>**Examples**<br>- Girls have equal opportunities for competition as boys' teams<br>- Competitive matches, leagues or tournaments are organized regularly for girls' teams within our program<br>- Our program sets up competitive matches, leagues or tournaments with other teams/organizations for our girls' teams<br>- During mixed gender competitions, activities and rosters are designed in ways that are equitable to girls		
**c) Skill Development**	**Score (1-5)**	**Notes and Comments**

Our organization gives girls regular opportunities to develop their sports skills and measure their development as they progress.<br>**Examples**<br>- Practices include teaching technical elements of the sport<br>- Sport activities are adapted to different skill levels<br>- Sport activities increase in difficulty as players build competency<br>- Sport activities are held with sufficient frequency (2 day/week or more) to develop skills<br>- Participants demonstrate competency in basic sport skills (dribbling, passing, understanding of rules, etc.) within the first year of participation		
**(d) Team Size**	**Score (1-5)**	**Notes and Comments**
Our program maintains an average daily attendance of 10 or more participants.<br>**Examples**<br>- The group size is sufficient to create a dynamic, meaningful, interpersonal team environment. Girls feel like they are a part of something bigger than their role.<br>- If a mixed-gender team, girls/boys representation reaches a 40/60 balance<br>- For games, there's consistently enough girls to field a full team		
**(e) Program accommodates for individual differences**	**Score (1-5)**	**Notes and Comments**
Our program ensures that all girls meaningfully participate in sports activities regardless of their abilities. We accommodate for cultural and linguistic diversity, as well as different education and literacy levels.<br>**Examples**<br>- Coaches and facilitators come from a variety of cultural and language groups<br>- An assortment of sports activities ranging from fun participatory play to competitive sports are offered to girls with different abilities and interests		
**1.4 Safe Spaces**		
**(a) Quality Playing Space**	**Score (1-5)**	**Notes and Comments**
Our organization provides an adequate playing space (court, field) for the level of competition that girls are involved in.<br>**Examples**<br>- The quality of the girls' playing space is comparable to the boys' playing space<br>- Cultural and religious considerations specific to girls are respected when organizing playing spaces<br>- Facilities are accessible to all girls, including girls with disabilities		
**(b) Safe Activity Spaces**	**Score (1-5)**	**Notes and Comments**

Our organization takes measures to ensure that activity spaces are emotionally, physically and developmentally safe for girls.<br>**Examples**<br>- The girls are physically safe at the field<br>- Bystanders support girls while they are playing rather than make them feel embarrassed, unsafe or watched.<br>- The girls are open to trying new sport skills and keep trying even if they don't get it right at first<br>- The girls feel like the team space is for them: they feel valued and like they can contribute		
**(c) Mixed Gender Activities**	**Score (1-5)**	**Notes and Comments**
Our program ensures that any mixed gender activities are planned and implemented in ways that prioritize girls' safety, inclusion, enjoyment and development.<br>**Examples**<br>- Activities avoid including a small number of "token" participants by ensuring a substantial number of both males and females participate<br>- Similar levels of skill and physical development is ensured across the group<br>- Leadership roles are divided intentionally and fairly among participants<br>- No rules that can be perceived as "charity" or "sorry rules" are applied to any group<br>- Mixed gender programs have at least one female coach represented<br>- There are consistent opportunities for girls to connect in girl-only specific spaces<br>- Additional steps are taken to ensure girls' ongoing comfort and happiness level with the team dynamic, such as individual check ins or home visits		
**(d) Toilets/Changing Rooms**	**Score (1-5)**	**Notes and Comments**
Our organization ensures that all girls have access to a safe and private place, where they can change and use the toilet before and after (sport) sessions, that is not accessible to boys or male staff/coaches while in use.<br>**Examples**<br>- Girls have their own dedicated toilets and/or changing rooms<br>- If changing rooms are shared, boys and men are not permitted in changing rooms during girls' activities		
**(e) Safeguarding Policies**	**Score (1-5)**	**Notes and Comments**

Our program has policies and protocol to assure the safeguarding of youth that participate in our program.<br>**Examples**<br>- Participants and their families/guardians are familiar with our safeguarding policies and sign consent forms<br>- All youth in the program have an adult that can confide in to talk through issues that come up on and off the field<br>- Coaches hav emergency plans in case incidents or injuries happen during activities<br>- Each activity - especially those which involve travel - have a safety plan for all participants<br>- We activively create a space where all stakeholders feel comfortable raising questions		
**1.5 Girls Leadership**		
**(a) Leadeship Roles**	**Score (1-5)**	**Notes and Comments**
Our organization provides girls with formal and informal leadership roles that matter and that provide a space for girls to grow new skills, contribute to culture and use their voices.<br>**Examples**<br>- Leadership roles could include captain, equipment manager, coach assistant, referee, or representing the program at an event<br>- Informal leadership roles could include leading stretches, sharing back in a circle, or being a welcomer to a new player on the team<br>- If the team is mixed gender, leadership opportunities given to girls avoid “token” roles that are more symbolic and aren’t respected by the boys		
**(b) Role Models**	**Score (1-5)**	**Notes and Comments**
Our organization ensures girls have access to diverse role models that promote playing sport and being a leader in the community.<br>**Examples**<br>- Female guest speakers, former or current female athletes<br>- Female staff members, especially senior managers and leaders<br>- There are team trips to watch women’s sports matches		
**(c) Decision Making**	**Score (1-5)**	**Notes and Comments**

Our program provides girls with opportunities to provide meaningful input into program design and implementation.<br>**Examples**<br>- Girls help to determine the scheduling of programming activities<br>- Girls decide the level of competitiveness of sports activities<br>- Girls provide input into the life skills content that is important to them<br>- Girls provide input into hiring new coaches		
**(d) Cultivating Leaders**	**Score (1-5)**	**Notes and Comments**
Our program identifies girls' leadership potential and works to support female talent development.<br>**Examples**<br>- Girls are told expectations and responsibilities of leadership roles<br>- High performing girls are identified and cultivated to grow along a leadership pathway with increasing responsibility<br>- There are multiple leadership opportunities that exist throughout the program for girls to show and strengthen their leadership skills (ex: leading a drill, taking attendance, etc.)		
**(e) Girl-led**	**Score (1-5)**	**Notes and Comments**
Our program provides girls with opportunities to give input on the community engagement strategy and lead activities that engage the community.<br>**Examples**<br>- Girls help decide on the groups of stakeholders that need to be involved in community engagement activities<br>- Girls take visible leadership roles in community meetings and events<br>- Our organization puts on special events to raise awareness of the program and to recruit girls for participation		
**1.6 Community of Support**		
**(a) Promotion**	**Score (1-5)**	**Notes and Comments**

Our program has developed and is implementing a strategy to promote the girls' sport program in the school-site's community if applicable (teachers, counselors, school-staff, etc.), and also among wider community partners (after-school programs, Parks & Recs groups, etc.).<br>**Examples**<br>- Past participants, female coaches and other female role models promote the program in their communities<br>- Parents are engaged in conversations about the benefits of sports for girls School/Partners/Community leaders are involved in supporting and promoting the program<br>- School/Partners/Community leaders are informed about and encouraged in promoting the program		
**(b) Attitudes of Boys & Men**	**Score (1-5)**	**Notes and Comments**
Within our program, the boys and men demonstrate allyship towards girls and women.<br>**Examples**<br>- Male coaches and coaches of boys teams attend games and other special events of the girls program<br>- Boys can play comfortably with girls; neither laughing at each other if a girl is more skilled than the boys nor isolating her or ignoring her for being a girl<br>- The boys know and use the names of the girls in the program<br>- Men in the program support women coaches to secure safe spaces and address unsafe bystanders on the field as needed		
**(c) Embededness**	**Score (1-5)**	**Notes and Comments**
Our program is valued, supported, and rooted in the community where we work. Key stakeholders like alumni, school officials, family, and community leaders are champions for the girls teams.<br>**Examples**<br>- Alumni return to their teams to coach or attend special events<br>- A network of supporters are available to fill gaps when needed- drivers, volunteers, sign makers, snack providers, etc.<br>- Family members, peers and/or teachers attend games to show support<br>- Family or friends bring food to team celebrations<br>- Staff and coaches are invited and attend community events, such as graduation and award ceremonies		
**(d) Advocates**	**Score (1-5)**	**Notes and Comments**

Enrollment and impact data collected by our program is filtered by gender and is visible to all relevant stakeholders (coaches, coordinators, key partners).<br>**Examples**<br>Youth enrollment by gender is shared with stakeholders<br>Results of youth surveys are disaggregated by gender and shared with stakeholders<br>Stakeholders understand why data is collected and how it's used		
**1.6 Continuous Learning**		
**(a) Access to Data**	**Score (1-5)**	**Notes and Comments**
Enrollment and impact data collected by our program is filtered by gender and is visible to all relevant stakeholders (coaches, coordinators, key partners).<br>**Examples**<br>- Youth enrollment by gender is shared with stakeholders<br>- Results of youth surveys are disaggregated by gender and shared with stakeholders<br>- Stakeholders understand why data is collected and how it's used		
**(b) Use of Participant Information in Program**	**Score (1-5)**	**Notes and Comments**
The demographic information collected by our program is used to improve our understanding of participants and to adapt programming when relevant.<br>**Examples**<br>- If data shows many new speakers of a specific language group, the program seeks a volunteer or family liaison who also speaks that language<br>- If data shows there are no 9th graders on a high school team, staff considers ways to improve 8th grade recruitment and retention strategies<br>- If data shows a participant is much older than their school grade, staff considers that student may have had interrupted formal education (SIFE)<br>- If there are no emergency contacts listed, that may indicate the player has few or no trusted adult supports		
**(c) Understanding Exits**	**Score (1-5)**	**Notes and Comments**

Our organization responds to girls exiting with curiosity and utilizes exit information to re-engage girls that do exit, advance recruitment efforts and shape program design.<br>**Examples**<br>- If a girl says that she's exiting the team, staff inquires why and if there's something that could make her change her mind<br>- If a girl exits and coaches can't connect with her, steps are taken to see if her friends know why she exited<br>- If there's a pattern around exits, coaches proactively ask more questions to understand this pattern and create an action plan that addresses reasons named by girls<br>- Exit data and reasons for girls' exits are logged<br>- Special attention is given when a girl exits a mixed-gender team		
**d) Youth Survey & Feedback**	**Score (1-5)**	**Notes and Comments**
Our program collects youth feedback through formal and informal means, analyzes the results, and acts to improve the program based on these youth inputs.<br>**Examples**<br>- Coaches ask girls for feedback about specific drills and games<br>- Coaches get girls' input when planning team building events or upcoming practice content<br>- Staff and coaches use multiple indicators to understand girls' experience (daily attendance, social media posts/engagement, smiling vs frowning faces)<br>- Coaches use barriers to entry named in feedback to make program design adjustments<br>- Coaches use feedback about youth experience to evolve team culture year over year		

On the field	Average		
Recruitment of Girls	Recruitment Plan & Goal		
	Active Recruitment		
	Multiple Entry Points		
Barriers to Regular Participation	Economic & Time Constraints		
	Adequate Equipment		
	Appropriate Apparel		
	Menstruation Management		
	Transportation /Travel		
Sport Program Quality	Coaching		
	Competitions		
	Skill Development		
	Team Size		
	Program accomodates for individual differences		
Safe Spaces	Quality Playing Space		
	Safe Activity Spaces		
	Mixed Gender Activities		
	Toilets/Changing Rooms		
	Safeguarding Policies		

On the sidelines	Average		
Female Coaches	Coach Recruitment		
	Female Presence		
	Equal Coaching Opportunities		
Access to Resources	Programming Resources		
	Equal Remuneration		
	Coaching Apparel		
Coach Leadership	Leadership Skill Development		
	Role Models		
	Coach Development Pathways		
	Visibility		
Coach Training	Technical Training		
	Gender Sensitive Coaching		
	Support & Feedback		
Coach Monitoring	Tracking Coaches		

In Organizations	Average		
Gender Strategy & Leadership	Gender Inclusion Strategy		
	Organiztional Gender Equity		
	Visibility of Female Leaders		
Gender Policies	Gender Inclusion Policy		
	Equal Opportunities Employment Policy		
Training of Personnel	Gender Training		
	Advancement Opportunities for Female Coaches/Facilitators		
	Advice		
Financial Resources	Balance of Financial Resources		
Communication & Advocacy	Gender Equity in Media Coverage		
	Training for Communications Staff		
	Gender Sport Advocacy		
Culture & Mindset	Support for Gender Equity		
	Positive Discrimination		
	Grievance		
	Willingness to Chnage		

Girls Leadership	Leadership Roles												
	Role Models												
	Decision Making												
	Cultivating Leaders												
	Girl-led												
Community of Support	Promotion												
	Attitudes of Boys & Men												
	Embeddednes s												
	Advocates												
Continuous Learning	Access to Data												
	Use of Participant Information in Program												
	Understanding Exits												
	Youth Survey & Feedback												
