# Supplementary material for: An exploratory evaluation of a shared learning community of practice approach to improving gender equity in sports and physical activity programming
Source: Front Sports Act Living. 2026 Sep 15;8:1895135. doi: 10.3389/fspor.2026.1895135 (PMC13619867; doi:10.3389/fspor.2026.1895135)
Supplement: Supplementary file 2 [file Datasheet2.pdf]

SLAT Exit Interview Guide

1) Starting with the assessment tool, walk me through (at a high level) your experience with the shared learning model and process?

2) What were your organization's goals for the SLAT process?
    a) If we don't know the stated goals from the GAP, probe for details.
    b) If we do know the stated goals, ask follow ups about goals achievement.

3) What were the biggest challenges and barriers participating in the SLAT process?

4) What were the biggest facilitators in participating in the SLAT process?

5) What was the most impactful part of the SLT process for you? For your organization?

6) If the Coalition does a model like this again, what should they keep in mind?
