# Supplementary material for: An exploratory evaluation of a shared learning community of practice approach to improving gender equity in sports and physical activity programming
Source: Front Sports Act Living. 2026 Sep 15;8:1895135. doi: 10.3389/fspor.2026.1895135 (PMC13619867; doi:10.3389/fspor.2026.1895135)
Supplement: Supplementary file 3 [file Datasheet3.pdf]

Supplement 1: Gender Action Plan for One Organization

GENDER ACTION PLANNING				
	**PRIORITY 1- On the Pitch**	**PRIORITY 2- On the Sidelines**	**PRIORITY 3 - In the Organization**	**PRIORITY 4**
**PRIORITY AREAS** Identify the priority area that needs improvement.	Active recruitment of at least 20 African American and African Immigrant girls ages 7 to 15 years old	Gathering Resources for both programming, participants and coaches	Training of Personnel and coaches	Provide girls specific physical development conversation around menstruation management.
**WHAT** What exact activities will you undertake to address this priority? Be specific.	Host a girls only information and team building activity session and get input on the type of physical activity that interests them.	Seeking grants. Seeking in-kind donations of sports apparels and equipment for coaches and girl participants.	Female coaches will develop leadership skills particularly important for sport coaching.	Host virtual learning sessions with 20 girls.<br><br>Provide them with resources like tampons and pads.
**WHEN** When will you start working on them? How long will it take?	Begin working on this by May 1, 2022 to May 30, 2022	Begin June 1, 2022 to July 30,2022	Begin In August 1, 2022 to October 31, 2022	Begin in July-November 30, 2022
**WHO** Who in the community should/could be involved? What are their roles?	The parents and girls participants. Parents are responsible for ensuring barriers directly related to girls' home responsibilities are removed. Girls will be encouraged to invite their friends to the event. Staff and coach	Program Administrator and coach seeks in-kind donations from sports stores and organizations	Administrator, coaches, doing outreach for partnerships and mentorship opportunities	Health Department and healhtychild.org website for resources, female elders in the community, parents, female coaches,<br>Girl participants

**HOW**<br>How will you deliver on your goals?<br>What support and resources do you need?	Funding for transportation, Recognition of girls who recruit other girls	Look to other cohort members for ideas and information	Making ourselves available for training, coaching, forming partnerships	Resources from Health department on girls body development, female elders from girls culture
**MEASUREMENT**<br>How will you measure your progress?	Feedback from girls and parents, roster, sign-in sheet	Number of positive responses received from donors.	Sign-in sheet, feedback, survey	Survey, Feedback, Sign-in sheet, Attendance
